# Progress in Biodegradable Flame Retardant Nano-Biocomposites

**DOI:** 10.3390/polym13050741

**Published:** 2021-02-27

**Authors:** Zorana Kovačević, Sandra Flinčec Grgac, Sandra Bischof

**Affiliations:** Department of Textile Chemistry and Ecology, Faculty of Textile Technology, University of Zagreb, Prilaz baruna Filipovića 28 a, 10000 Zagreb, Croatia; zorana.kovacevic@ttf.unizg.hr (Z.K.); sflincec@ttf.unizg.hr (S.F.G.)

**Keywords:** bioplastics, biocomposites, nanobiocomposites, biodegradability, flame retardancy, bast fibres

## Abstract

This paper summarizes the results obtained in the course of the development of a specific group of biocomposites with high functionality of flame retardancy, which are environmentally acceptable at the same time. Conventional biocomposites have to be altered through different modifications, to be able to respond to the stringent standards and environmental requests of the circular economy. The most commonly produced types of biocomposites are those composed of a biodegradable PLA matrix and plant bast fibres. Despite of numerous positive properties of natural fibres, flammability of plant fibres is one of the most pronounced drawbacks for their wider usage in biocomposites production. Most recent novelties regarding the flame retardancy of nanocomposites are presented, with the accent on the agents of nanosize (nanofillers), which have been chosen as they have low or non-toxic environmental impact, but still offer enhanced flame retardant (FR) properties. The importance of a nanofiller’s geometry and shape (e.g., nanodispersion of nanoclay) and increase in polymer viscosity, on flame retardancy has been stressed. Although metal oxydes are considered the most commonly used nanofillers there are numerous other possibilities presented within the paper. Combinations of clay based nanofillers with other nanosized or microsized FR agents can significantly improve the thermal stability and FR properties of nanocomposite materials. Further research is still needed on optimizing the parameters of FR compounds to meet numerous requirements, from the improvement of thermal and mechanical properties to the biodegradability of the composite products. Presented research initiatives provide genuine new opportunities for manufacturers, consumers and society as a whole to create a new class of bionanocomposite materials with added benefits of environmental improvement.

## 1. Introduction

**Figure 1:**
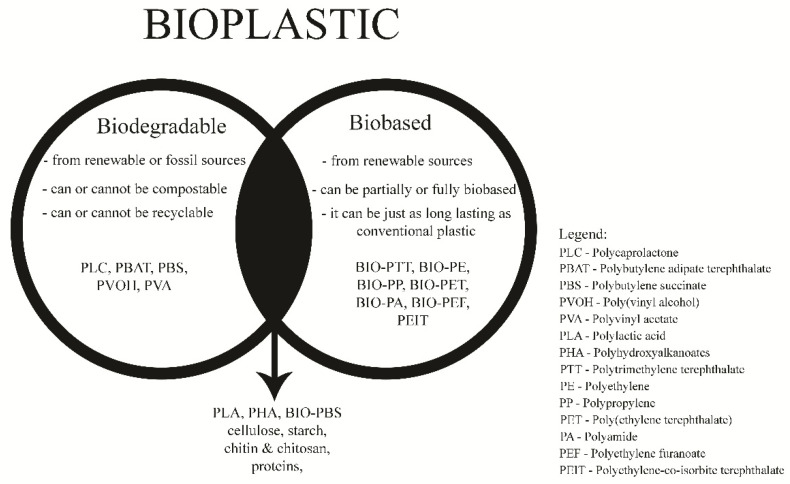
Bioplastic and differentiation between the groups of biobased and biodegradable polymers.

With continuous growth for more than 50 years, global plastic production reached 368 million tonnes in 2019 [1]. Consequently, economic and environmental sustainability is questioned, since fossil resources are still extensively used in plastic production. The development of bioplastics from biological or annually renewable resources could alleviate the huge environmental problem caused by the enormous plastic waste disposal at landfill space and marine environment [2,3,4]. According to the association European Bioplastics (EUBP), bioplastics are defined as plastic materials which are either biobased, biodegradable or feature both properties [5]. From the beginning, it is necessary to distinguish the term biodegradable from the term biobased. Biobased properties are connected to the product’s origin, while biodegradable properties tackle the product end-of-life issues (Figure 1).

According to the programme of Biobased Industries (BBI), it is expected that ~30% of fossil based raw materials will be replaced by biobased and biodegradable ones by 2030. Expectations are that two-thirds of the global chemical industry will eventually be based on renewable resources [6,7].

Nowadays, the terms “bio”, “eco”, “green” and “sustainable” are of great importance and novel technologies strive to incorporate them in their development strategies. One such technology is the production of composite materials, in particular biocomposite production where fibre-reinforced composites enter the ecological niche of the multibillion market. Due to the strict requirements for most of the products used in public areas, one of the major functionalities of developed biocomposite is its resistance to fire. The main drawback of biocomposites reinforced with lignocellulose material is their susceptibility to combustion when exposed to heat flux or a flame source. Therefore, the role of flame retardant (FR) products is to reduce negative consequences caused by flames, contact and radiant heat, sparks, molten metal dripping and hot gases and vapours [8]. The challenge of the usage of solely ecologically-benign FR agents is even more demanding. For a very long time the most popular FR agents were halogen based, but today more stringent legislation initiated the development of a new generation of nanosized FR agents. Although there is a broad review in the literature on FR agents, a literature search revealed no previous studies that have been conducted regarding environmentally benign nanosized FR agents. Therefore, this review will put the main emphasis on the biodegradable composites reinforced with natural plant fibres modified with FR nanofillers and the specific problem to be addressed within this paper is regarding the sustainability of FR treatments. We hypothesized that the current stage of nanotechnology development only partially satisfies environmental issues, therefore it is possible to replace conventional FR treatments with biodegradable ones. The forthcoming development of novel nanobiocomposites, introducing multifunctionality, represents a hot topic in which the application of nanotechnology and biodegradable polymers will open up new opportunities for the improvement of properties and the cost-price-efficiency.

## 2. Biodegradability of Biocomposites

**Figure 2:**
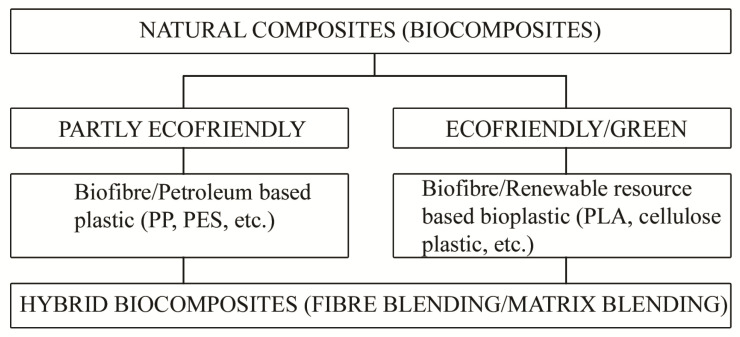
Classification of natural composites or biocomposites [9].

Although biocomposite materials have been known from ancient times, in recent years there has been a growing interest in the research and development of composite materials that are biobased and/or biodegradable and, moreover, that show structural and functional stability in storage and usage [6]. Biocomposites consisting of natural reinforcements, for example, vegetable fibres, can be: partly eco-friendly with non-biodegradable conventional polymer matrix, for example, polypropylene, polyethylene, epoxy, polyester, and so forth, or they can be fully eco-friendly with a biodegradable polymer matrix, for example, soy, starch, cellulose, PLA, PLC, PBS, and so forth. Biocomposites made from plant fibres and biopolymers are more environmentally friendly biocomposites and are called “green” composites (Figure 2) [6].

Fully biodegradable biocomposites exhibit biodegradability and/or compostability properties [6,10,11,12]. The properties of currently used biobased and biodegradable polymers are shown in Table 1.

Table 1 represents the wide application of biobased and biodegradable polymers, although it is often necessary to alter their features by combining various additives, by their mixing or by manufacturing composite materials made of them.

Bioplastics reinforced with biofibres represent a new biocomposite that, in many applications, substitute the composite materials reinforced with glass fibre and, in most cases, meet strict requirements of European Union (EU) Directives. Natural fibre reinforcements with the addition of specific modifiers, that is, coupling agents, cross-linking agents, flame retardant agents, antimicrobial agents and so forth, are able to improve composite final properties, such as mechanical and flame-retardant properties and fire resistance, as well as water and gas barrier properties [11].

The examples of the already known applications of biocomposites made from bast fibres, such as flax, hemp or *Spartium junceum* L., are in the automotive and construction industry [21,22]. The most commonly used fibres for wide applications are the natural plant fibres presented in Table 2 [23].

Bast fibres are popular because of their commercial availability and sustainability, which makes them desirable for reinforcements in polymer composite materials production. They are abundantly available, fully and easily recyclable, non-toxic, biodegradable, non-abrasive to the moulding machinery, easily coloured and have a lower cost, lower density and lower energy consumption in the producing phase, as compared to synthetic fibres such as glass and carbon fibres [8]. Additionally, bast fibres are shatter resistant, have good sound abatement capability, non-brittle fracture on impact, high specific tensile modulus and tensile strength, low thermal expansion coefficient and low mould shrinkage. Bast fibres are obtained from the outer cell layers of the stems of various plants and are constituted of cellulose, hemicellulose and lignin [8,88,89,90,91]. The main plants used for the supply of bast fibres are flax, jute, hemp, ramie and kenaf. These types of fibres have a lower lignin and higher cellulose content than wood fibres. The cellulose in bast fibres also tends to be more crystalline (80–90%) than that of wood fibres (50–70%) [92]. Higher cellulose and lower lignin content are responsible for the improvement of mechanical properties, therefore tensile properties of composite materials reinforced with bast fibres are mainly improved since natural fibres show higher strength and stiffness values in comparison with matrix polymers.

The interest in the use of plant fibres, especially lignocellulosic bast fibres, as reinforcement in polymeric composites is steadily increasing because of the continuously higher demands for environmental regulations and ecological concerns of global society. Biodegradability starts to be one of the producers’ major concerns and boosts new design processes.

By definition, biodegradation is the chemical breakdown of materials into smaller compounds in physiological environment, using microorganisms such as bacteria and fungi. Biodegradable plastics undergo microbially induced chain scission, leading to mineralization, photodegradation, oxidation and hydrolysis, which can alter a polymer during the degradation process [93]. Many bioplastics are conceived to be biodegradable, and some of them are conceived to be compostable. Compostable bioplastic materials break into smaller compounds in a specific timeframe in a controlled moist, warm, aerobic environment to produce compost that is non-toxic and can enhance soil and support plant life. For a polymer to be categorized as compostable, the following four criteria must be fulfilled according to EN 13432 [94,95]:

1. Disintegration—namely fragmentation and loss of visibility of the compostable material in the finished compost. It is measured in a pilot composting test (EN 14045) in which specimens of the test material are composted with biowaste for 3 months. After this time, the mass of test material residues has to amount to less than 10% of the original mass.
2. Biodegradability—namely the capability of the compostable material to be converted into CO_2_ under the action of microorganisms. The standard contains a mandatory threshold of at least 90% biodegradation that must be reached in less than 6 months (laboratory test method EN 14046) [96].
3. Ecotoxicity—the amount of heavy metals has to be below given maximum values. The final compost must not be affected negatively (no reduction of agronomic value and no ecotoxicological effects on plant growth)
4. Absence of negative effects on the composting process.

Generally, all bioplastics and conventional plastics will biodegrade at some point, but many of them need hundreds of years, while producing toxic residues at the same time. A smaller but steadily growing group of compostable bioplastics biodegrade within a specific timeframe under clearly defined conditions (e.g., temperature, humidity and the presence of microorganisms).

### Biodegradation Mechanism

Biodegradation is usually a one-step process using only biological activity, while a small number of polymers show a two-stage decomposition process, where heat also plays an important role. Biodegradation can take place under oxygen conditions—aerobic biodegradation (1) or within the conditions where oxygen is not available—anaerobic biodegradation (2).

In aerobic biodegradation, organic matter is oxidized leading to the transformation of carbon (C) to carbon dioxide (CO_2_). This conversion asks for the consumption of oxygen (O_2_) through which carbon of the sample is converted into carbon dioxide and water. Some of the carbon can remain as a residual sample or in metabolites, representing the total residual carbon, while some of the carbon is used to produce new biomass.

C_sample_ + O_2_ → CO_2_ + H_2_O + C_residual_ + C_biomass._ (1)

In anaerobic biodegradation there is no consumption of oxygen. The sample is converted into methane (CH_4_) and a CO_2_, residual sample or metabolites and biomass. Anaerobic conditions are created when oxygen is not present or when oxygen is consumed or depleted more rapidly than it is replaced (mostly by diffusion) [93,97].

C_sample_ → CH_4_ + CO_2_ + C_residual_ + C_biomass._ (2)

The primary indicator of biodegradation is the production of CO_2_ and/or CH_4_, the consumption of O_2_, while the secondary effects of biodegradation are visual disappearance, weight loss, decrease in molecular weight, and so forth and they refer to incomplete biodegradation.

Rate and degree of biodegradation are determined by various factors, which can differ from one environment to another. These factors are moisture content, oxygen availability, temperature, type and the amount of used microorganisms (bacteria, fungi) and enzymes, as well as salt concentration [98].

Kovačević, Z. et al. (2019) [99] found out that the biocomposite material made of biodegradable PLA polymer and reinforced with natural fibres (*Spartium junceum* L.) showed a positive degradation effect based on its weight loss, while using 50 wt.% proteases enzyme (serine endopeptidase) concentration during a five day treatment. These natural fibres were previously modified with montmorillonite nanoclay (MMT) and citric acid (CA), which most likely affected the biodegradation rate of such composite material regarding the presence of excess –OH groups. These groups may accelerate the hydrolytic decomposition responsible for higher biodegradation of composite samples [100]. The role of CA in this research was in crosslinking natural fibres with polymer matrix and MMT. While forming the composite material, CA melted and was incorporated into cellulose chains, breaking the intra- and intermolecular hydrogen bonds. It influenced “minor” cleavage of cellulose chains and, as a consequence, more rapid biodegradation of material reinforced with these fibres resulted.

**Figure 3:**
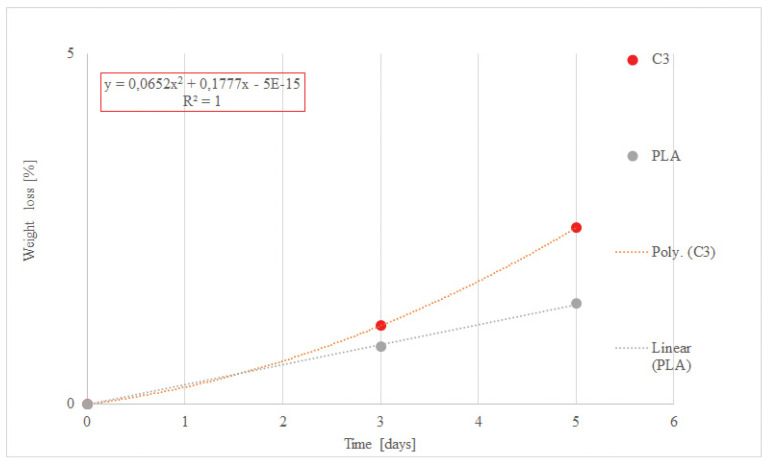
Linearity and polynomial regression of weight loss/time function for neat PLA and its natural fibres reinforced (NFR) composite during the degradation using 50% of enzyme Savinase 16 L, where PLA is neat polylactide polymer, and C3 is composite made of PLA and 3F fibres (*Spartium junceum* L. fibres modified with montmorillonite nanoclay (MMT) and citric acid (CA)).

According to Figure 3, which indicates reliability in the data of the used trendlines regarding R squared, composite material will degrade by a minimum of 90% weight loss within 6 months of the biodegradation treatment, which is one of the requirements in EN 14046. More accurately, composite material (C3) will degrade within 36 days, while neat PLA will degrade by the minimum of 90% weight loss within 315 days. In conclusion, each material that shows a biodegradation effect, such as losing its weight for a minimum of 90% in the period of 180 days and converting it to CO_2,_ will be declared as biodegradable.

The increasing use of natural fibres reinforced (NFR) biocomposites provides a better and healthier life for every individual, and the steady improvement of our eco-system. Additionally, NFR biocomposites reveal advanced properties like antimicrobial and water resistant properties, as well as flame retardancy, especially if specific measures are taken considering its biodegradation at the end of the product life time.

## 3. Flame Retardant Chemistry 

FR chemistry of composites is highly complex and there is no universal approach to flame retardancy. For this reason, scientists most often choose flame retardants based on the chemistry of polymer thermal decomposition and fire hazard scenarios. Additionally, there is a whole list of commercial requirements imposed on materials, such as price, processing method, colour, environmental stability, together with the most recent ones—sustainability and recyclability [101].

Despite numerous positive properties of natural fibres, the flammability of plant fibres is one of the most pronounced drawbacks for their wider usage in biocomposites production.

Flame retardancy of natural fibres depends mostly on their chemical composition, such as cellulose, hemicellulose, lignin, pectin and wax content, as well as on their crystallinity and orientation.

Hemicellulose and cellulose start decomposing within the temperature range of 200–260 °C and 260–350 °C, respectively. Char, volatiles and gases that is, CO, methane and ethylene are produced during thermal decomposition. Within a temperature range of 280–350 °C, levoglucosan is formed. With a further increase of temperature, decomposition results in flammable volatiles, gases and carbonaceous char. Lignin is thermally decomposed within the temperature range of 160–400 °C. Bond breakage occurs at a lower temperature, while at the higher temperature the cleavage of bonds in the aromatic rings occurs. The formation of an insulating char layer helps to protect the fibre from oxidation.

The orientation of fibres within the polymer matrix is another feature that can influence flammability by controlling the fibre permeability to oxygen [102,103]. For instance, a layer with randomly oriented fibres is often more permeable to oxygen than a layer with unidirectional or bidirectional oriented fibres. Because of higher flammability, which biocomposites made of plant fibre and polymer matrix often show, chemical modifications are considered to diminish these drawbacks.

The usage of cellulose fibres as reinforcement in biobased polymers leads to higher flammability of NFR composite materials. In general, cellulose based polymers decompose at 300–500 °C into gas and condensed phases, producing combustible gases, liquids, char and smoke with dripping that could be hazardous.

**Figure 4:**
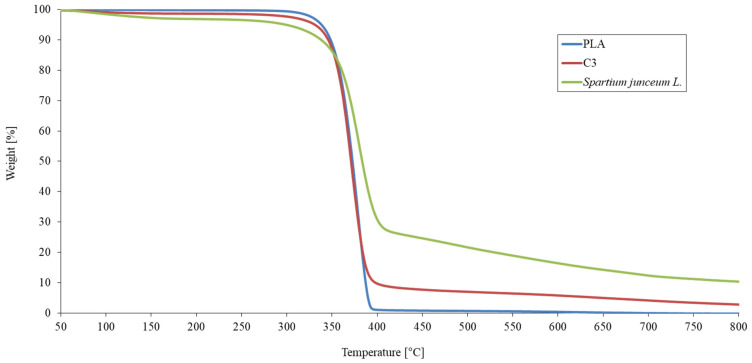
TGA curves of pure PLA, *Spartium junceum* L. fibres and nanobiocomposite (C3) made of PLA, *Spartium junceum* L. fibres and MMT nanofillers crosslinked with citric acid.

In the paper [56] published by Kovacevic, Z. et al. (2015) the authors presented the improvement of the nanobiocomposite thermal properties when natural fibres were modified with nanoclay in the presence of citric acid as a crosslinker (Figure 4). The initial decomposition temperature of nanobiocomposite material (C3) slightly decreased from 354 °C (for the pure PLA) to 350.5 °C while the second decomposition temperature at 60% weight loss decreased from 376.5 °C (for the pure PLA) to 374.6 °C.

Although the thermal decomposition of nanobiocomposite started earlier by addition of FR nanofillers, it resulted in producing more char yield compared to the neat PLA. Alves, J. L. et al. (2020) have investigated the flammability of PLA polymer treated with organo montmorillonite nanoclay modified with a mixture of surfactants based on ammonium and phosphonium salts. The addition of organoclay nanofillers decreased the initial degradation temperature of PLA at 5 wt.% of material mass loss from 329 °C for the pure PLA to the 314 °C for the PLA nanocomposite treated with organoclay modified with dialkyl ester dimethyl quaternary ammonium ion. Although the same trend was already reported in the literature while using MMT nanofillers and PLA polymer [104], this very same sample of nanoclay with 8 wt.% loading shows reduced flammability, which is visible by the reduction of peak heat release rate (PHRR) for 38% [105].

Chemicals, for example, citric or phytic acid, may activate cellulose hydroxyl groups or introduce new moieties that can effectively influence fibres and/or matrix regarding their better flammability properties. The most common modification treatments are alkaline, acetylation, benzoylation, peroxide, isocyanate, silane, grafting, coupling agents and nanoparticle treatments [11,106,107,108,109,110,111,112].

One of the outstanding drawbacks of macrosized FR agents is their high loading rate in the composite material of 40–70%, which negatively influences composite mechanical properties. Nano FR agent is dispersed in one of the phases of composite material. It has been already presented through the extensively published literature that nano FRs significantly reduce heat release rate, they retard ignition and decrease the speed of flame propagation with the addition of only 2–10% of nano FRs to the total material weight [113].

### 3.1. Nanofillers Classification and Chemistry

Chemical modifications of natural fibres and/or biopolymers with nanosized fillers open a new perspective for biocomposite materials with special incidence in environmentally friendly materials (nanobiocomposites), including food packaging materials and materials used in biomedical fields, for example, drug-delivery, biosensors, cancer diagnosis and tissue engineering [114]. Research on nanobiocomposites can be considered a new interdisciplinary field closely related to significant markets, such as automotive and construction engineering.

**Figure 5:**
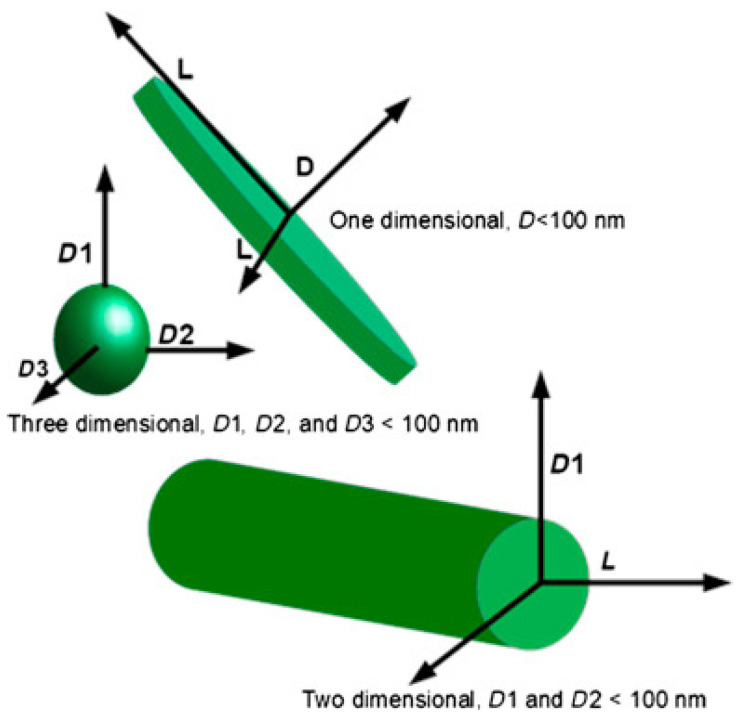
Classification of nanofillers. Reprinted from Polymer Composites with Functionalized Nanoparticles: Synthesis, Properties, and Applications Micro and Nano technologies, Akpan, E. I., Shen, X., Wetzel, B., Friedrich, K., Chapter 2 -Design and Synthesis of Polymer Nanocomposites, Pages No. 47–83, Copyright (2019), with permission from Elsevier.

The production of nanobiocomposite materials implies the usage of three components: convenient matrix that is usually biobased, reinforcement from renewable sources and modification fillers (nanofillers Figure 5), which have at least one dimension (length, height or width) less than 100 nm (e.g., nanotubes, nanofibers, clay nanoparticles, hydroxyapatite and metal nanoparticles, nanocellulose crystals, and so forth) [115].

By using nanosized fillers, nanocomposites can significantly improve mechanical, thermal, barrier and physico-chemical properties, when compared with pure polymers and conventional composites reinforced with microsized fillers [116,117]. Fibres are usually modified with the following nanofillers: TiO_2_, ZnO, Ag, Au, SiO_2_, Al_2_O_3_ [118,119,120]. There are different structures of nanofillers such as nanorods, nanoflowers, nanodiscs, nanospheres, and so forth. Their geometrical characteristics may affect the properties of the treated material [121,122,123,124,125].

Table 3 presents most commonly used nanofillers for the improvement of composite properties [4]. 

### 3.2. FR Nanofillers

**Figure 6:**
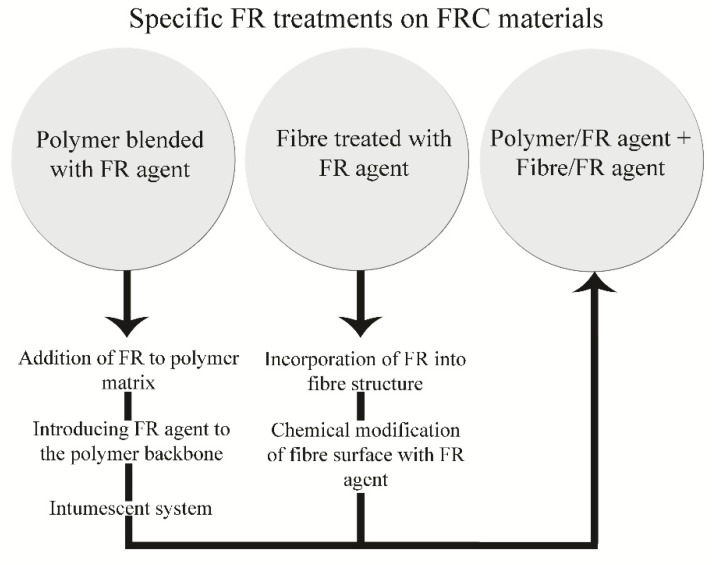
Specific flame-retardant treatments on fibre reinforced composite materials.

A novel method for the FR improvement of polymer composite properties is FR nanofiller treatment. FR nanofillers are nano sized flame retardants, which can be easily incorporated into the composite system in the way presented in Figure 6.

Nanofillers can be blended with a polymer, which is quite simple and effective process, or they can be chemically introduced into the polymer structure.

Another method of polymer FR treatment is the usage of an intumescent system. Intumescents are widely used as additives for polymers acting as char promoters.

**Figure 7:**
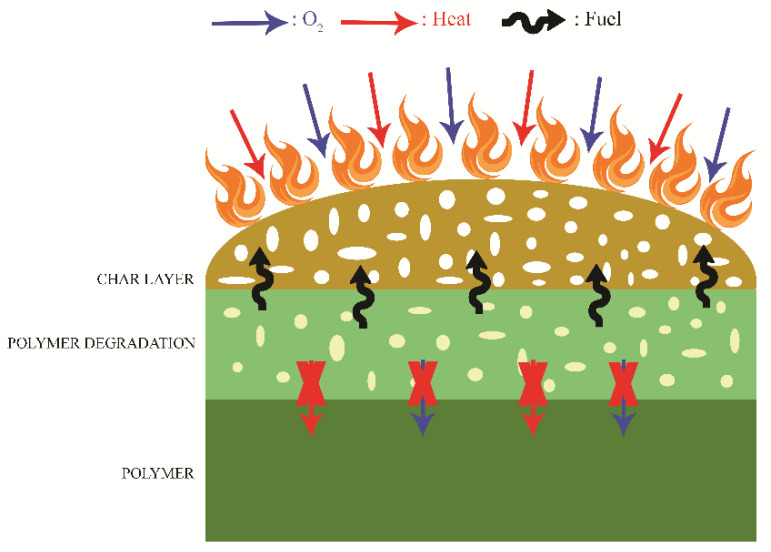
Mechanism of an intumescent system.

The intumescent FR mechanism is more effective and is environmentally friendlier, due to the fact that char forming systems incline to the abortion of the burning cycle prior to the flame poisoning. Intumescent flame retardants influence the expanding and swelling up of treated material, which results in the formation of a char protective layer at the material surface (Figure 7). This layer limits the oxygen diffusion to the site of combustion and therefore protects the treated material [126,127].

The intumescent system consists of three equally important components:

1. Acid source—dehydrate carbohydrates which forms a char layer. Its quantity depends on the number of carbon atoms and reactive hydroxyl or carboxyl sites [128].
2. Carbonizing agent—should have high thermal stability to maintain polymer processing and contain hydroxyl or carboxyl functional groups responsible for char formation—cellulose, starch, alginates, lignin, chitosan, tea saponin, and so forth [126,129].
3. Blowing agent—decomposes and releases gas during thermal decomposition of the carbonizing agent in order to expand carbonized layer [128].

Additionally, such FRs do not need a high loading concentration. Usually, less than 20 wt.% is required and even less if nanofillers are used as carbonizing agents.

Different techniques can be used to add nanofillers to fibres—the pad-dry-cure method or impregnation process, layer-by-layer (LbL) assembly, plasma treatment, wet chemical etching, hydrothermal treatment, vapor deposition, sol-gel method, application of a synthetic binder or electroless deposition. Chemical or mechanical binding of nanofillers to the surface of fibres is done with the aim of improving compatibility between fibres and polymer [130,131].

Recently, it seems that the incorporation of FR nanofillers during the final surface finishing processing of fibres has evolved into an interesting approach. The main advantage is that a low loading concentration can be used. Not all the commercially available nanofillers can be used in the flame retardancy field. It was confirmed that several key points need to be considered in order to achieve improved results. The shape and size of the nanoparticles are directly related to processing conditions, chemical nature, concentration, as well as their distribution as a function of the applied processing method [131].

The incorporation of nanofillers into biocomposite systems in order to decrease their combustibility and flammability is one of the recent flame-retardant modifications. Flame retardancy proves to be one of the most important safety properties required of the materials to be used for transport, construction, military and aerospace purposes.

**Figure 8:**
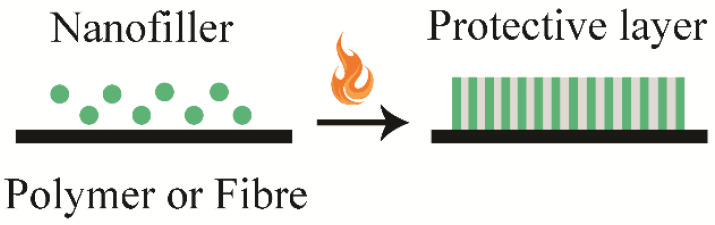
Formation of protective residue layer and char formation on the surface of material.

Two main general mechanisms of flame retardancy have been noticed in the case of nanobiocomposites decomposition—the formation of a physical barrier and a catalytic charring effect (Figure 8) [132]. The physical barrier and the catalytic charring effect reduce the heat release rate during polymer burning [126,133,134].

Figure 8 presents an inorganic or organic protective residue layer, which isolates treated material from the heat flux of the flame, reduces heat transport as well as volatiles transport into the flame zone. This protective layer should be dense to make the FR effect as good as possible; therefore, a good dispersion of nanofiller is extremely important [102].

**Figure 9:**
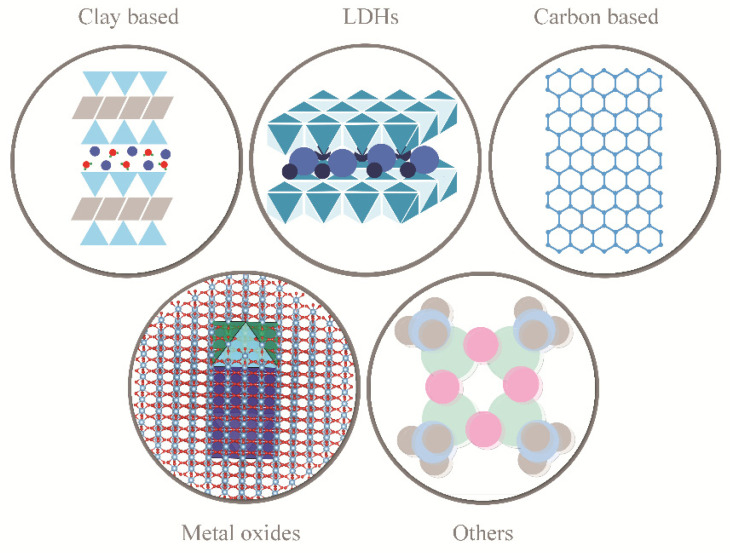
Most frequently used nanoflame retardants (nano FRs): clay based, layered double hydroxides (LDH), carbon based, metal oxides and other.

The most commonly used nano flame retardants in the nanobiocomposite production are presented in Figure 9 [135,136,137].

#### 3.2.1. Clay Based Nano FRs

Nanoclays are one of the most commonly used flame retardants for reducing the flammability of materials. These nanofillers are layered silicates which are frequently used in the synthesis of nanocomposites, because of their availability, versatility and respectability towards the environment and health [138]. Most clays are smectite layered silicates consisting of 2 tetrahedral layers sandwiching 1 octahedral layer [139]. Since the forces between the layers are weak, small organic molecules are often introduced between them [140,141]. The benefits of nanoclay usage as modifiers in the material processing include their low cost, and overall improvement of mechanical, thermal, electrical and optical properties of the end-products. FR mechanisms for silicate clay containing nanocomposites are radical trapping and barrier mechanism [142]. Table 4 shows different usages of clay based nanofillers utilized for flame retardant properties.

It can be noticed from Table 4 that all clay based nanofillers offered a decrease in the flammability of the tested materials. Fire sensitivity parameters were reduced by more than 30% for the peak heat release rate (PHRR) and 10% for total heat release (THR). There are different types of geometry in clay based nanofillers, which may influence flame retardancy of such nanocomposite materials. Yang, F. and Nelson, G.L. (2011) have investigated synergistic effect of different geometry nanofillers and NASA developed an FR agent (SINK). They have combined fume silica (spherical) with attapulgite nanoclay (rod-like) FR nanofillers and gain a positive synergistic effect with SINK, which was visible as a remarkable reduction in heat release rates of polystyrene (PS) composites. For instance, the introduction of 20% SINK into PS reduced the PHRR of polystyrene for 31%; 10 wt.% silica reduced it for only 13%, while the combination of silica and SINK reduced it for 56%, which clearly shows synergistic effect of nanosilica and SINK [149]. Isitman, N.A. et al. (2012) have investigated the influence of a nanofiller’s geometry on the flame retardancy of PLA polymer. PLA nanocomposites were additionally treated with aluminium diethylphosphinate (AlPi), which is microsized commercial FR agent with trade name Exolit OP 1240. Plate like nanofiller (MMT nanoclay) showed an improvement in flame retardancy over spherical and rod like nanofillers. Its PHRR was reduced for 50% compared to the neat PLA [150]. It is also important to emphasize that nanodispersion of nanoclays have major influence on the positive flame retardancy properties. Both the above mentioned papers [149,150] explained good results with better exfoliation of nanoclay inside the polymer influencing its larger surface area and thus rapid migration and accumulation of its platelets or spheres on the exposed surface forming dense and intact char layer thus establishing an effective barrier to heat and mass transfer prior to intumescent char formation. In the paper published by Ye, L. et al. (2016), it can be noted that the usage of just one type of FR is often not enough to achieve satisfying FR properties. The presence of AlPi smooths the way for melt intercalation of PLA into the organo modified MMT nanoclay leading to a more exfoliated nanocomposite structure where 3 wt.% of organo modified MMT nanoclay and 17 wt.% of AlPi reduce PHRR by 26.2% compared to the neat PLA while showing LOI of 28% [151]. From the literature overview presented in Table 4, it is visible that the combination of clay based nanofillers with other nanosized or microsized FR agents can improve the thermal stability and FR properties of nanocomposite materials. Limiting Oxygen Index can be above 30%, while under a laboratory UL 94 vertical burning test it is possible to show the V-0 rate. Green FR materials have a tendency to incorporate commercial FRs recommended to be used as environmentally friendly FRs such as Mg(OH)_2_, ammonium polyphosphate (APP) or expandable graphite (EG) in the composite system. Since microsized commercial FRs need to be added in composite system in higher amounts of up to 60 wt.% causing negative impact on the material’s mechanical properties it is desirable to achieve enhanced FR properties by synergistic activity with much lower FR concentrations of up to 10 wt.%. Clay based nanofiller loading in the Table 4 was in the rage 0.5–10 wt.%; however, it is interesting to note that biodegradable PLA needed higher loading from 3% to 10%, regardless of whether the treated material was neat PLA polymer or PLA composite reinforced with natural fibres [24,56,143,144,147]. Sypaseuth, F.D. et al. (2017) have achieved satisfying flammability properties of PLA nanocomposites by synergistic activity of commercial Mg(OH)_2_ FR agent and 5 wt.% of nanoclay visible as a reduction of PHRR for 42% but without any significant improvement in the material’s mechanical properties [152].

Although, the authors have done a broad literature review on the topic of FR nanofillers and most of the papers refer to the improvement of overall composite properties while using a combination of microsized and/or nanosized FR agents, a lot of research is still needed in optimizing the parameters of FR compounds to meet all the requirements, from the improvement of thermal and mechanical properties to the biodegradability of such material and at the same time to the biodegradability of the nanofiller incorporated in the composite material.

#### 3.2.2. Layered Double Hydroxides

Layered double hydroxides (LDHs) show a layered structure consisting of positively charged brucite-like layers while interlayer region contains charge compensating anions and solvation molecules. Their structure enables flexibility and adjustable chemical composition as well as high anion exchange capacity, which allows for their usage as flame retardants. They are potentially eco-friendly flame retardants for polymer applications. The FR mechanisms of LDHs consists of the reduction of fuel accessible for combustion, thus reducing fire intensity, improves the formation of carbon-based layer due to the char formation, and improves the stabilization of char and the dilution effect [142]. Table 5 shows the usage of LDHs as FR agents for neat polymer and natural fibre treatment.

It can be noticed from Table 5 that LDHs effectively reduced negative fire sensitive parameters. Loading of LDHs nanofillers is similar to clay based nanofillers, within the range of 0.1–10%. Total heat release was reduced by more than 10%, while peak heat release rate was reduced by more than 50%, using only 5% loading of LDHs. Positively charged layers of LDHs consist of divalent and trivalent metal cations such as Mg^2+^, Zn^2+^, Al^3+^, and so forth. Different metal types in the LDH structure influences flame retrdancy of polymer/LDH composite material. Wang, D-Y. et al. (2010) have investigated the flammability of PLA nanocomposite materials. The authors used two different LDHs, which differ in metal cations used (Mg^2+^ or Zn^2+^). PLA treated with 2 wt.% MgAl-LDH shows a decrease in THR for 24% while PLA treated with 2 wt.% ZnAl-LDH shows a decrease in THR of 30% compared to neat PLA [161]. LDHs, like any other nanofillers, will agglomerate when loaded in higher concentrations, therefore modification of LDHs is a good decision to improve its dispersion within composite material. Additionally, modification of LDHs with other FRs can lead to the synergistic effect of FR components, thus improving the flame retardancy of composite material. Barik, S. et al. (2017) applied MgAl-LDHs on the cotton fabric without any modifications in order to improve UV protection, mechanical and flame retardancy properties. LOI value of the cotton treated with 1.5 wt.% of LDHs was 20.8%, which pointed to the unsatisfactory FR treatment since FR textile should have an LOI higher than 25% [155]. Such disadvantages could be improved by modifying LDH or by mixing it with other FRs. Kalali, E. et al. (2015) have modified LDHs with a complex of several modifiers. Cyclodextrin (CD) influences the formation of rich char residues while sodium dodecylbenzensulfonate (DBS) and taurine (T) acting as a dispersion and crosslinking agent, respectively. Only 6 wt.% of modified LDH have an increased LOI value to 26.8% and shows instantaneous extinguishing compared to pristine epoxy that has LOI 23% [162]. However, by mixing more than two FRs, a synergstic effect occurs and a strong char layer is formed during the burning process. Gao, Y. et al. (2018) have investigated the flame retardancy of PP treated with ammonium phosphate (APP) intercalated LDHs and zinc borate (ZB) and 10 wt.% of APP-LDH combined with 2 wt.% of ZB decreased PHRR for 42% compared to pristine PP [163]. A paper published by Xu, S. et al. (2020) [159] showed much higher nanofiller loading due to the usage of FR complex made of hydrotalcite LDH intercalated with sodium alginate (SA) into its interlayer space followed by melt blending with a PP polymer. Since the sodium alginate is mixed with LDH in the 1:1 composition, the LDH loading is twice as small and indicates a positive synergistic effect of this complex on the FR properties of PP polymer, which is evident from the reduction of the PHRR and the THR by 69% and 9%, respectively. Moreover, more than two metal cations inside the LDHs will improve the flammability properties of polymer matrix which is presented in the paper published by Wang, B. et al. (2019). Intercalated CaMgAl-layered double hydroxides were added to the acrylonitrile-butadiene-styrene resin (ABS) and 8 wt.% of modified three metal LDHs was combined with 1.5 wt.% of APP and 23 wt.% of expandable graphite (EG) in order to achieve a desirable FR effect. The three metal LDH nanocomposites formed a stable and compact char layer which lead to the improvement of ABS composite flame retardancy visible as an increase in the LOI value to 28.8% [164].

#### 3.2.3. Carbon Based Nano FRs

Carbon based FRs have become popular due to their non-toxicity and environmentally benign properties. There are several different carbon-based materials which show FR behaviour (graphene, carbon nanotubes (CNTs), expandable graphite, and so forth). They can increase polymer thermal stability and reduce the heat release rate. However, the flame retardancy of polymer or their composite materials is not significantly improved if the carbon based nanofillers are used alone. Therefore, they need to be mixed with other FR materials to achieve a better performance [165,166]. Table 6 shows the usage of carbon-based nano flame retardants for the treatment of materials consisting of neat polymer and/or natural fibres. The loading of nanofillers is in the range of 0.1–10%.

It can be noticed from Table 6 that carbon based nano FRs are generally used in synergism with other micro and/or macroscopic size FRs in order to improve material flammability properties. Higher loading of carbon based nanofillers can be noticed in the paper published by Schartel, B. et al. (2003) [167]. The usage of 25 wt.% of EG shows an increase in LOI of 43% compared to polypropylene/flax composites without the addition of any FRs. Expandable graphite (EG) is intumescent FR agent and it requires relatively higher loading to be efficient in flame retardancy unless it is combined with other types of FRs. In this case, EG was used alone in the PP/flax composite material and due to its enormous expansion, an endothermic combustion reaction was developed in view of carbon oxidation, which reduced flames owing to oxygen consumption. The expansion created a heat barrier layer and dripping reduction as well. It could be concluded from Table 6 that graphene oxide (GO) nanofiller is, in most cases, used in synergism with other compounds and nanofillers containing phosphorus, nitrogen, silicon and boron elements such as nanoclay, chitosan, polyaminoamides, ammonium molybdate, and so forth. Tawiah, B. et al. (2019) have investigated synergistic effect of azo-boron (AZOB) modified reduced graphene oxide (RGO) intercalated by sodium metaborate (SMB) on flame retardancy and smoke/toxic fumes suppression. Only 1 wt.% of such GO modified nanofiller have reduced PHRR for ~58%, THR for ~46%, total smoke release ~21%, and peak CO_2_ production by ~60%. A very good V-0 rating was attained in the UL 94 test with a higher LOI value of 28.6% [178]. Besides EG and GO, both multi-walled carbon nanotubes (MWCNTs) and single-walled carbon nanotubes (SWCNTs) are used as FR nanofillers for a wide range of polymers [179]. Kashiwagi, T. et al. (2006) have compared the same loading (0.5 wt.%) of SWCNTs and MWCNTs nanofillers regarding the rheological response of the poly(methyl methacrylate) (PMMA) nanocomposite material. SWCNTs shows a much higher elastic response than MWCNTs influencing polymer viscosity as well. An increase in polymer viscosity has a positive effect on flame retardancy by slowing down the emission of combustable volatiles, avoiding polymer dripping and by mechanically stabilizing the charred structures. SWCNTs have a much larger surface area per unit volume and thus a higher interfacial area with the polymer matrix leading to a compact char layer [180]. Like any other carbon based nanofiller, carbon nanotubes provide better FR effects if combined with other compounds, especially with nanoclays [144]. In recent years there has been a great interest in using biochar as a flame-retardant agent. The paper published by Barbalini, M. et al. (2020) [177] presented a flame-retardant system based on bio based products—phytic acid and biochar. Biochar is the carbon-rich byproduct obtained from biomass carbonization. Although the particle size of biochar in this paper was microscopic, it was possible to produce nano biochar by fixing the pyrolyzing temperature, by an exfoliation process or by a ball milling process [181].

#### 3.2.4. Metal Oxides

Metal oxide nanoparticles are also commonly used as flame retardants because of their low toxicity and low cost [182,183]. They are generally obtained by a bottom-up and top-down approach. The bottom-up approach includes physical methods such as pulse and physical vapour deposition, chemical vapour deposition, pulsed laser deposition, atomic layer deposition, spray pyrolysis, and so forth; and chemical methods such as reduction reactions in water-based media, sol-gel, electrochemical, microemulsions, and so forth are used; biological methods include bioreduction by plants, bacteria, fungi, yeast, algae, and so forth. Top-down approach includes bulk nanomachining, nanomilling, spark erosion, lithography, and so forth. [184]. Similar to other nanofillers, metal oxide nanoparticles show the best properties when used in combination with other FRs.

Table 7 presents the usage of metal oxide nanoparticles as FRs during neat polymer and/or natural fibre treatment. It is interesting that among all reviewed FR nanofillers in this paper, metal oxides are considered as the most commonly used nanofillers for textiles, especially ZnO and SiO_2_ nanoparticles. Sheshama, M. et al. (2017) have investigated the flame retardancy of sisal yarns when nano ZnO was incorporated inside. Although synergism with other FRs is more efficient regarding improvement of FR properties, in this work, the authors have used only nano ZnO and they have achieved an excellent result for LOI of 34% [185]. Metal oxides gather on the exposed surface creating a protective barrier layer. This inert layer segregates oxygen from the combustible gases and suppresses smoke as well. A good example of the synergistic effect of nano ZnO on the intumescent FR system (IFR) was presented in the paper published by Rao, T. et al. (2020). The addition of IFR composed of APP and N-Ethanolamine Triazine-Piperazine, Melamine Polymer (ETPMP) to the neat epoxy resin resulted in an increase in LOI for 35% compared to pristine epoxy. Further, by adding nano ZnO to this system resulted in an even greater increase in LOI value in proportion to the increase of nanofiller loading. Respectively, 1 wt.%, 2 wt.% and 3 wt.% of nano ZnO showed an LOI 30.2%, 32.7% and 34.2% [186]. Most often the loading rate of such nanofillers is in the range 0.01–10%. The lowest loading of metal oxide nanoparticles was presented in the paper published by Samanta, K.K. et al. (2017) [187]. Even 0.01% of nano ZnO particles imparted high fire retardant properties, which was visible through the increase of the LOI value to 35%.

In the paper published by Gallo, E. et al. (2013) [188], a double-component laminate was developed for balancing fire retardancy and mechanical performance of composite materials. The FR layer consisted of a phosphorus-based FR and 2% of a nano size metal oxide that was on the top of kenaf fibres. Natural lignocellulose fibres are rich in hydroxyl groups and are often used as a carbonization agent, forming porous structure inside the fibre that affects the release of pyrolysis gases. An improvement of flammability properties was visible through significant reduction of the PHRR (47%).

Treatment of cotton fabric with SiO_2_ nanoparticles by using the hydrothermal method was explained in the paper published by Zhou, T. et al. (2020). Cotton fabric was firstly immersed into the PEPAS and CYPA solution [189], for which formulations are visible in Table 7, and was then subsequently immersed in a sodium silicate aqueous solution and sealed in a high pressure reactor at 160 °C for 12 h. Flame retardancy of this material (TCFSi) was examined by LOI. The LOI value of TCFSi reached 31.8 and decreased to 27.8 after 20 launderings. The durability of metal oxide nanofillers on the cellulose textile materials is another important point of view. This segment is still insufficiently explored, thus is very important challenge for researchers.

#### 3.2.5. Other Flame Retandant Nanofillers

Considering the European Green Deal and other global ecological strategies, the environmental issues are emphasized and new ecological treatments are proposed. Green flame retardants according to the EU and Stockholm convention should meet several principles, as presented in Table 8 [197].

Among the already listed FR agents, there are other commonly used bio-sourced FR nanofillers like calcium carbonate, cyclodextrines, lignin, proteins, hydroxyapatite and silsesquioxanes [198]. Table 9 presents the usage of such additives with the aim of flammability properties’ improvement. The loading rate of these nanofillers is in the range 1–10%. The usage of nano calcium carbonate yields better flammability properties when combined with polyphosphates, due to its intumescent behaviour [199,200]. Carbohydrates, such as cyclodextrin nanosponges and nano lignin, seem to be very useful components in flame retardant systems [201,202]. Lignin is seen as an efficient bio-based carbonization agent in intumescent systems. Chollet, B. et al. (2019) [203] prepared lignin nanoparticles from Kraft lignin microparticles by dissolution-precipitation process, followed by phosphorus grafting onto the nanoparticles. Phosphorus grafting at the lignin surface confirmed their effective usage as FRs, even at a low loading concentration (5 wt.%).

Polyhedral oligomeric silsesquioxanes (POSS) have the smallest size compared to other nanofillers and can be easily incorporated into different polymers, at the same time enabling improved mechanical properties and reduced flammability [204]. Turgit, G. et al. (2018) investigated flame retardancy of polymer composites based on combination of intumescent FR and POSS. Only 0.5 wt.% of POSS is needed to impart high FR properties of such composites, while the addition of 1 wt.% POSS combined with 19 wt.% of intumescent FRs show a huge reduction in PHRR and THR values, by 33% and 32%, respectively [205].

Nano hydroxyapatite is a calcium phosphate bio-filler present in bones and shells. It has been used to improve flammability properties of different polymers like polycarbonate, cellulose, polyvinyl alcohol, but it has also been the most commonly used as the additive in the PLA polymer, where the highest loadings of HA (10% and more) have been noticed [206,207]. Khalili, P. et al. (2019) published the paper where the high loading of nano HA imparted good thermal resistivity to the composite material made of PLA and flax fibres while inducing its lower mechanical properties at the same time [207].

Table 4, Table 5, Table 6, Table 7, Table 8 and Table 9 present very broad applications of different FR nanofillers which could be used not only for polymer but generally for fibre treatment as well. These nanofillers exist in a variety of structures and shapes and exhibit low or no toxicity, which is very important from the point of view of possible biodegradability and/or compostability, especially taking into consideration the ecotoxicological effects on the soil and future plant growth. Although low- or non-toxic and environmentally benign FR nanofillers are presented in this review, it is important to note the toxicity of polymer composite materials treated with such nanofillers, especially in view of the emission of toxic smoke when burning. The toxic smoke of flaming composites consists of combustion gases, char particles and tiny fragments, which can cause serious health problems. Generally, polymers with an aliphatic backbone will generate a lower amount of smoke than polymers with pendant aromatic groups [214]. Carbon monoxide (CO), carbon dioxide (CO_2_) hydrogen cyanide (HCN), hydrogen chloride (HCl), nitrogen oxides (NOx), sulphur dioxide (SO_2_), aldehydes, polycyclic aromatic hydrocarbons (PAHs) and so forth, are the most common gases developed during thermal decomposition and combustion of polymeric materials [215]. Since FRs can act in the condensed (polymer breakdown, charring, intumescence) and/or in the gas (gas dilution, chemical quenching of active radicals) phase it is worth noticing that flame inhibition through the gas phase dramatically increases the yield of CO, smoke and toxic gases [216,217]. The most common mechanism of FR nanofillers is increasing char yield during composite thermal degradation, which provides a good barrier to prevent the transfer of heat and volatiles, thus consequently reducing smoke formation [218]. For this reason, FR nanofillers often have a positive impact on the reduction of overall fire hazards, not only on flammability but on toxic gases and smoke development as well. Hassan, M. et al. (2016) investigated the effect of MMT nanoclays on the flammability parameters of PE composites and concluded that only 1% of MMT decreased CO and CO_2_ emission [219]. LDHs show a high level of smoke suppression which can be seen in the paper published by Xia, W. et al. (2021) through the analysis of the smoke production rate (SPR), total smoke production (TSP), total smoke release (TSR) and the production of CO and CO_2_. All of these parameters were reduced, especially TSR, which was decreased by 67.2% at the combustion stage, indicating lowering of toxic smoke production [220]. Bensadoun, F. et al. (2011) used 3 wt.% MMT as nanofiller in the environmentally friendly resin and got a reduction of 66% in smoke density [221]. In the paper published by Bajwa, D.S. et al. (2019), the addition of 1 wt.% crystal nanocellulose and nano ZnO particles (CNC-ZnO) lowered the total smoke release by 9%, compared to pure HDPE [193]. Sometimes the best effect considering smoke suppression is achieved when nano FRs are used in synergism with nano smoke suppression agents, which was described in the paper published by Zhou, K. et al. (2016). It was noticed that carbon based nano FRs in combination with a nano smoke suppression agent significantly reduced evolved CO by 45%, compared to pure polymer [214]. Another example of a positive smoke suppression property was presented in the paper published by Qu, L. et al. (2020), where graphene oxide sheets were functionalized with POSS and were incorporated in the epoxy resin. Only 0.7 wt.% of such nanofiller affected the decrease in total smoke release by 41.5% [222].

Another positive effect of nano FRs is related to very low concentrations of such nanofillers. A lower concentration of nanofillers loading positively affects the value added features and, quantitatively, fewer chemicals are used. It can be seen that each of the Table 4, Table 5, Table 6, Table 7, Table 8 and Table 9 shows improvements of fire sensitivity parameters (peak of heat release rate, total heat release, heat release capacity, smoke density, total smoke production, and so forth), as well as the increase of LOI value and thermal stability, where both groups exhibit improved FR properties.

## 4. Conclusions

The growth of the nanotechnology industry has affected scientific, technical and economic competitiveness of polymers and composites based on renewable sources in the development of a range of high-performance engineering and consumer products. More recently, researchers have been investigating the usage of nanostructures, such as cellulose nanostructures, carbon nanotubes, nanoclays and so forth as reinforcing elements to obtain a new class of nanobiocomposites. Fundamental properties of nanoparticles wereadded to improve the thermal, mechanical, optical, electrical and many other functional properties of composite materials were usually opposed to the environmental requirements of sustainability and biodegradability.

The novel trend of producing biodegradable materials in order to protect our resources from extinction and to leave something to our descendants as a legacy, is gaining popularity nowadays. The most commonly used biodegradable composite materials are made of a polymer matrix and natural fibre reinforcement, capable of degrading within a 6 month period by at least 90% of their initial weight. The usage of such biodegradable materials is highly extensive and they are most commonly used in the automotive and construction industries. While designing and producing such materials, the user should always be in the centre of the designer’s thinking and the emphasis should be on his health and safety. One of the most important safety aspects is the preparation for the event of fire and the prevention of possible fatal casualties. During fire, a significant release of heat and smoke could cause serious damage to humans and a huge loss of their property as well. As a significant amount of the products on the open market belong to the group of biocomposites today, it has become important to guide the design of biocomposites in such a way as to combine flame retardant functionality with environmental requirements. Therefore, conventional biocomposites have to be altered through different modifications to be able to respond to the stringent standards posed on automotive and construction industries.

Flame retardant treatments of one or all the components of biocomposites are crucial to overcoming the burning deficiency and extending the applicability of widely used natural fibre reinforced composites. Recently, there has been a great interest in the use of FR nanofillers that can effectively stop the burning process (heating, decomposition, ignition, combustion and flame propagation) of natural fibre composites. Considering the vision of the future, which is described with concepts such as green, eco, sustainable and so forth, there is a strong demand for FR nanofillers to be biodegradable as well. A new type of composite, called nanobiocomposites, has emerged and opened a door to the implementation of advanced, high performance, lightweight green nanocomposites, as a replacement for conventional non-biodegradable petroleum-based plastic materials.

Current concerns about the development of biodegradable FR nanobiocomposite materials focus on the fulfillment of Green Plan goals that requires not only the application of exclusively environmentally friendly agents but zero waste as well. Furthermore, what is biodegradability rate of FR nanofillers is still insufficiently explored, especially when they are combined with other microsized FR agents. Therefore, the problem of fully environmentally benign nanofillers’ usage is still present. These findings lead us to future prospects of the wider usage of various plant cultures, especially energy crop cultures such as *Miscanthus x giganteus* or *Sida hermaphrodita* as valuable sustainable sources for NFR composites within BIOCOMPOSITES project, while at the same time developing biodegradable FR nanofillers to impart flame retardancy in nanobiocomposite material.

## Figures and Tables

**Table 1:** Properties of the most commonly used biobased and/or biodegradable polymers [10,13,14,15,16,17,18,19,20].

Biobased Polymer	Description
PLA (Polylactide)	Renewable, biocompatible and biodegradable polymer. Obtained by ring opening polymerization of lactide or by direct polycondensation of lactic acid. Its thermal stability and impact resistance are inferior to those of conventional polymers used for thermoplastic applications. PLA matrix is used to improve the composite stiffness, permeability, crystallinity, and thermal stability. Targeted markets include packaging, textiles and biomedical applications.
PHA (Polyhydroxyalkanoates)	Renewable, biocompatible and biodegradable polyesters synthesized by microorganisms from various carbon sources. They are very sensitive to temperature and shear. Additives, blends and natural fibre reinforced composites are capable of overcoming negative drawbacks of individual components.
STARCH	Bioplastic composed of both linear and branched polysaccharides (amylose and amylopectin). Thermoplastic starch (TPS) can be obtained from starch by disrupting its molecular interactions, using plasticizers and/or complex operations as devolatilization, melt-melt mixing and morphology control. Extreme moisture sensitivity of starch leads to limited practical application. Therefore, blending of TPS with other less sensitive polymers and additives is required.
CELLULOSE	Renewable, biocompatible and biodegradable polymers obtained from wood, cotton or extracted from agricultural byproducts such as bagasse, stalks and cropstraws. Cellulose based materials are used in two forms on an industrial scale—Regenerated cellulose used for fibre and film production and Cellulose esters used in coatings, biomedical uses and other usual plastic applications.
CHITIN & CHITOSAN	They are renewable, biocompatible and biodegradable polymers with excellent adsorption properties. Chitin is a natural polysaccharide used as a supporting material in many invertebrate animals such as insects and crustaceans. The deacetylated chitin is known as chitosan. Chitosan has been explored for films and fibres and has generated great interest and usage for biomedical applications.
PROTEINS	They are biodegradable polymers based on renewable sources obtained from original proteins which can be classified as plant and animal proteins. Water, glycerols, fatty acids and oils are commonly used plasticizers for proteins. Wet and dry processing methods are used to obtain biomaterials from proteins. Such biomaterials are used in food and pharmaceutical applications, as well as in tissue engineering applications.
BIO-PBS (Bio-Polybutylene succinate)	Renewable, biodegradable and even compostable material obtained by direct polymerization of biobased succinic acid and 1,4-butanediol. This material is mainly used for product containers and packaging since it is food-contact approved.
PBAT/PLA (Poly(butylene adipate-co-terephtalate)	Polybutyrate is a biodegradable and compostable biopolymer with properties similar to low density polyethylene (LDPE). PBAT bioplastic is made from fossil resources. Its compounds (starch, PLA) have a biobased carbon content of up to 30%. Typical application is for flexible film for packaging, e.g., compostable shopping bags.

**Table 2:** Plant fibres used as reinforcement in polymer matrix.

Plant Fibre	Matrix	Source
Flax	Starch, PBT, PP, PLA	[24,25,26,27,28,29,30]
Hemp	Epoxy, PBS, PP	[31,32,33,34,35]
Jute	Epoxy, PP, PLA	[36,37,38,39,40,41,42]
Kenaf	PLA, PET, PP	[43,44,45,46,47,48,49]
Ramie	PCL, PBS, PP, Starch, Epoxy	[50,51,52,53,54,55]
Spanish Broom	PLA, PP	[56,57,58]
Sisal	Bioepoxy, PES,	[45,59,60,61,62,63]
Coir	PLA, starch, Epoxy, PP, PE	[64,65,66,67,68,69]
Banana	PVA, PP, Epoxy, PU	[70,71,72,73,74,75,76]
Bamboo	Starch, PLA, Epoxy	[60,77,78,79,80,81,82]
Miscanthus Giganteus	PP, PLA, PBS/PBAT	[83,84,85,86,87]

**Table 3:** Division of nanofillers by its dimensionality.

Plate Like (1D)	Nanofibres/Nanowhiskers (2D)	Nanoparticles (3D)
• Layered Silicates (Montmorilonite-MMT, Hectorite, Saponite)	• Carbon Nanotubes (Single-walled and Multi-walled)	• Silica Particles (SiO_2_)
• Layered Double HydroxIdes—LDHs	• Cellulose Nanofibrils	• Metal Oxides (TiO_2_, Al_2_O_3_, MgO, ZnO, Fe_2_O_3_, Fe_3_O_4_)
• Graphene Nano Sheets/ExPanded Graphite	• Cellulose Nanocrystals	• Metal Hydroxides (Nanomagnesium Hydroxide)
• Layered MoS2 Nano Sheets	• Bacterial Cellulose	• Metal Nanoparticles (Ag, Au, Cu, Fe)
• Layerd Nano α-Zirconium Phosphate	• Sepiolite Nano Rods	• Polyhedral Oligomeric Silsesquioxane (POSS)
• Pseudo-Boehmit (AlOOH)	• Halloysite Nanotubes	• Fullerene
• Black Phosphorus	• Gold or Silver Nanotubes	• Carbon Black
• MXenes Nano Sheets (Matal Carbides and/or Carbonitrides)	• Wormlike Rubber	• Spherical Nano Rubber
• Hexagonal Boron Nitride	• Boron Nitride Nanotubes	• Quantum Dots

**Table 4:** Clay based FR agents.

Matrix/Reinforcement	FR Agent	Loading	Flammability	References Year
PLA	Two Organo-modified Layered Silicates (OMLS)	3%	PHRR 42% ↓	[143] 2010
PLA/Hemp	• Sepiolite Nanoclay • MWCNT	10% 2%	PHRR 45% ↓	[144] 2010
PU	• Chitosan • MMT Nano Clay	0.1% 1%	PHRR 52% ↓	[145] 2012
PLA/Spanish Broom	• MMT Nano Clay	5%	THR 13.5% ↓	[56] 2015
PP/Kenaf	• Halloysite Nano-tubes (HNTs) • Montmorillonite (MMT) Nanoclay	3% 3%	THR 20% ↓	[146] 2016
PLA	• Nano Fibrous Sepiolite SEP-DOPO	10%	UL-94 V-0	[147] 2019
PLA/Flax	• Sepiolite nNnorods • Chitosan • APP	1% 0.5% 1%	PHRR 33% ↓	[24] 2020
PP	• Nano Kaolin	1.5%	LOI 35.5%	[148] 2020

**Table 5:** Layered double hydroxides FR agents.

Matrix/Reinforcement	FR Agent	Loading	Flammability	References Year
PHB	• Organically Modified LDH Sodium Stearate	5%	THR 13.2% ↓	[153] 2012
Epoxy	• Eugenol Derivative Based LDH	8%	UL-94 V-0	[154] 2014
Cotton	• Mg–Al Nano-LDH	1.5%	LOI 20.8%	[155] 2016
Cotton	• Inorganic Hydrotalcite Nanoparticles (HT)	0.1%	THR 27% ↓	[156] 2017
Bamboo	• MgAl-LDH	5%	THR 33.3% ↓	[157] 2019
Silicon Rubber SR/PBS	• Mg_4.5_Al_2_(OH)_3_(CO_3_)_6_⋅5H_2_O (LDH)	5%	PHRR 54.4% ↓	[158] 2019
PP	• Sodium Alginates LDH (SA@LDHs)	30%	UL-94 V-0	[159] 2020
Leather	• LDH/Zanthoxylum Bungeanum Seed Oil	10%	LOI 28.3%	[160] 2020

**Table 6:** Carbon based FR agents.

Matrix/Reinforcement	FR Agent	Loading	Flammability	References Year
PP/Flax	• Expandable Graphite (EG)	25%	LOI 30%	[167] 2003
PLA	• Sepiolite Nanorods • Multiwalled Carbon Nanotubes (MWNT)	10% 2%	PHRR 45% ↓	[144] 2010
PP/Carbon fibre	• Carbon Black	5%	THR 16% ↓	[168] 2015
Polyimide PI	• Graphene Oxide Nanosheet (GO) • MMT	5% 10%	LOI 55%	[169] 2017
Epoxy/Fruit fibres	• EG	7%	THR 25.5% ↓	[170] 2017
PU	• Chitosan • GO	0.5% 1%	THR 13% ↓	[171] 2019
Epoxy/Curaua	• GO	0.1%	DTA Increase in Thermal Stability	[172] 2019
cotton	• Polyamidoamines Containing Disulphide Groups (SS-PAA) • Nano GO	12% 1%	PHRR 53% ↓	[173] 2019
ABS	• Mo5/PN-rGO	1%	THR 20% ↓ Total Smoke Production (TSP) 45% ↓	[174] 2020
Hydroxyethyl Cellulose (HEC) + Lactic Acid (LA) + PU	• Graphene Nanoplatelets (GNPs)	0.3%	TGA Enhancement in Thermal Stability by Nearly 15–20 °C With a Weight Loss of 50%.	[175] 2020
Elium^®^ 150 Thermoplastic Resin	• EG • Alumina Trihydrate (ATH)	4–10% 10–30%	UL-94 V-0	[50] 2020
PLA	• Carbon Nanotubes (CNTs) • CaMg-Ph	1% 19%	PHRR 35% ↓	[176] 2020
Cotton	• Phytic acid • Biochar	8% 8%	No Ignition	[177] 2020

**Table 7:** Metal oxide FR agents.

Matrix/ Reinforcement	FR Agent	Loading	Flammability	References Year
PMMA	• TiO_2_ • Fe_2_O_3_ • OMMT (Cloisite 15A)	10% 10% 10%	PHRR 29% ↓	[190] 2005
Kenaf/PHBV/ PBAT	• Exolit OP 1240 • Antimony Oxide NPs Sb_2_O_3_	8% 2%	PHRR 47% ↓	[188] 2013
Unsaturated Polyester PES	• Exolit OP 1240 • Nano Al_2_O_3_	10–15% 2.5%	UL-94 V-1	[191] 2015
Sisal	• Nano ZnO	1%	LOI 34%	[185] 2017
Jute	• Nano ZnO • Polyhydroxymethyl Amino Silicone (PHAMS)	0.01% 10%	LOI 35%	[187] 2017
Cotton	• Nano-TiO2@DNA	3%	Do Not Ignite	[192] 2019
HDPE	• Cellulose Nano Crystals + ZnO NPs	0.4%	PHRR 18% ↓	[193] 2019
Zinc Alginate (ZnAlg)	• Nano-cuprous Oxide (Cu_2_O)	/	LOI 58%	[194] 2019
Epoxy +PA	• Intumescent Fire-retardant (APP+pentaerythritol+Melamine) • Clamshells CS Bio Filler • Nano TiO_2_	52% 3% 1%	Smoke Density Rating (SDR) 37.5% ↓	[195] 2020
Cotton	• pentaerythritol phosphate Urea Salt (PEPAS) • 2-(4-(4,6-dichloro- 1,3,5-triazin-2-ylamino) phe- nylsulfonyl) ethyl sulphate sodium (CYPA) • nano-SiO_2_	300 g/L 125g/L /	LOI 31.8%	[189] 2020
Wood	• chitosan/sodium phytate/TiO_2_-ZnO nanoparticle	1%	LOI 32.8%	[196] 2020

**Table 8:** Legislation principles of greener flame retardance.

**Legislation principles of greener flame retardance**
not PBT (Persistent, Bio-accumulating, Toxic)
not POPs (Persistent Organic Pollutant)
REACH/CLP: No/Few Hazard (H) or Risk (R)
not CMR (Carcinogenic, Mutagenic, Toxic to Reproduction)
not EDC (Endocrine Disrupting)

**Table 9:** Other environmentally friendly FR agents.

Matrix/ Reinforcement	FR Agent	Loading	Flammability	References Year
Waste PP +Kenaf	• Nano CaCO_3_ • Sodium polyphosphate (NaPP)	7% 13%	PHRR 18% ↓	[199] 2012
PP PA PE	• Cyclodextrin Nanosponges	10%	THR 11% ↓	[208] 2012
Cotton	• POSS	20 bilayers (BLs)	PHRR 20%↓	[136,209] 2015
PP	• POSS • Intumescent Flame Retardant	0.5% 19.5%	UL 94 V-0 LOI 29.9%	[205] 2018
Epoxy/Kenaf	• Nano Oil Palm Empty Fruit Bunch (OPEFB) Filler	3%	LOI 30%	[210] 2019
PLA	• Lignin Nanoparticles diethyl (2-(triethoxysilyl) ethyl) phosphonate (SiP)	5%	PHRR 11% ↓	[203] 2019
PLA/Flax	• Nano hydroxyapatite Ca_10_(PO_4_)_6_ (OH)_2_	40%	UL-94 V-1	[207] 2019
PLA	• Nanoplate-like Hydroxyapatite (HA)	10%	TGA Improved Thermal Sstability	[206] 2020
epoxy	• Chicken Feather Nano HA From Conch Shells	15% 3%	HRC Heat Release Capacity 36% ↓	[211] 2020
PU	• HA • Sodium Alginate (SA) • Chitosan (CH)	1% 0.5% 0.5%	PHRR 77.7% ↓	[212] 2020
PAN	• Tannic Acid-MoS_2_ Nanosheets	2%	PHRR 38.1% ↓	[213] 2020

## Data Availability

The data presented in this study are available on request from the corresponding author.
